# Network reconfiguration and working memory impairment in mesial temporal lobe epilepsy

**DOI:** 10.1016/j.neuroimage.2013.01.036

**Published:** 2013-05-15

**Authors:** Pablo Campo, Marta I. Garrido, Rosalyn J. Moran, Irene García-Morales, Claudia Poch, Rafael Toledano, Antonio Gil-Nagel, Raymond J. Dolan, Karl J. Friston

**Affiliations:** a Faculty of Psychology, Autonoma University of Madrid, Madrid, Spain; b Wellcome Trust Centre for Neuroimaging, University College London, 12 Queen Square, London, UK; c University Hospital of San Carlos, Epilepsy Unit, Neurology Department, Madrid, Spain; d Hospital Ruber Internacional, Epilepsy Unit, Neurology Department, Madrid, Spain

**Keywords:** Hippocampal sclerosis, Working memory, Connectivity, Dynamic causal modelling, Temporal lobe epilepsy

## Abstract

Mesial temporal lobe epilepsy (mTLE) is the most prevalent form of focal epilepsy, and hippocampal sclerosis (HS) is considered the most frequent associated pathological finding. Recent connectivity studies have shown that abnormalities, either structural or functional, are not confined to the affected hippocampus, but can be found in other connected structures within the same hemisphere, or even in the contralesional hemisphere. Despite the role of hippocampus in memory functions, most of these studies have explored network properties at resting state, and in some cases compared connectivity values with neuropsychological memory scores. Here, we measured magnetoencephalographic responses during verbal working memory (WM) encoding in left mTLE patients and controls, and compared their effective connectivity within a frontotemporal network using dynamic causal modelling. Bayesian model comparison indicated that the best model included bilateral, forward and backward connections, linking inferior temporal cortex (ITC), inferior frontal cortex (IFC), and the medial temporal lobe (MTL). Test for differences in effective connectivity revealed that patients exhibited decreased ipsilesional MTL-ITC backward connectivity, and increased bidirectional IFC-MTL connectivity in the contralesional hemisphere. Critically, a negative correlation was observed between these changes in patients, with decreases in ipsilesional coupling among temporal sources associated with increases contralesional frontotemporal interactions. Furthermore, contralesional frontotemporal interactions were inversely related to task performance and level of education. The results demonstrate that unilateral sclerosis induced local and remote changes in the dynamic organization of a distributed network supporting verbal WM. Crucially, pre-(peri) morbid factors (educational level) were reflected in both cognitive performance and (putative) compensatory changes in physiological coupling.

## Highlights

► Effects of hippocampal sclerosis (HS) on effective connectivity were evaluated. ► HS induced a network adjustment in the ipsilesional and the contralesional hemisphere. ► Strength in ipsilesional and contralesional couplings was negatively related. ► Contralesional couplings were inversely related to performance and level of education. ► These findings support the notion that network alterations underlie cognitive decline.

## Introduction

The analysis of functional integration in the brain is not only useful to investigate normative cognitive processes, but also provides an unique window into neurological and neuropsychiatric disorders, as their clinical impact is related to the integrity of distributed neural networks (Bassett et al., 2009; Buckholtz and Meyer-Lindenberg, 2012; Guye et al., 2008). In the last decade, there has been an increasing number of studies using structural and/or functional connectivity methods to evaluate the clinical impact of medial temporal lobe epilepsy (mTLE) on neural networks (Bettus et al., 2009; Bonilha et al., 2012; Doucet et al., 2012; Frings et al., 2009; Liao et al., 2011; Liu et al., 2012; Vaessen et al., 2012; Vlooswijk et al., 2011a,b; Voets et al., 2012; Wagner et al., 2007; Yogarajah et al., 2008; Zhang et al., 2010). Hippocampal sclerosis is the most frequent pathological finding in patients with this form of epilepsy (Wieser, 2004). However, these studies have shown that connectivity abnormalities are not restricted to the pathological hippocampus, but can also be observed in other connected structures within the same hemisphere, or even in the contralesional hemisphere. These findings have led to the notion of mTLE as a ‘network disease’ (Bonilha et al., 2012). Most of the connectivity studies conducted with mTLE patients have explored network dynamics at resting state, and in some cases compared connectivity values with neuropsychological scores. In contrast, very few studies have investigated effective (directed) connectivity changes in mTLE (Addis et al., 2007; Vlooswijk et al., 2010b; Voets et al., 2009; Waites et al., 2006). We have previously characterized lesion-related changes in effective connectivity during WM performance, in a group of patients with mTLE associated with left hippocampal sclerosis (HS) (Campo et al., 2012). Our focus on WM was motivated by previous neuropsychological studies demonstrating impaired WM in patients with mTLE-HS, (Stretton and Thompson, 2012). Furthermore, neuroimaging studies have shown differences in brain activation during WM tasks, when mTLE patients were compared with healthy controls (Campo et al., 2009; Cashdollar et al., 2009; Stretton et al., 2012). Using dynamic causal modelling (DCM), we observed that the strength of specific connections involving the MTL was reduced in the ipsilesional hemisphere, while they increased in the contralesional hemisphere. Importantly, this enhancement was negatively related to task performance, signalling a reorganization process, operating with different efficiency across patients (Braun et al., 2008). These findings showed that WM impairment was linked to specific network changes, and suggest that certain patterns of reorganization could be related to functional compensation (Guedj et al., 2011; Maccotta et al., 2007; Powell et al., 2007; Tracy et al., 2012). However, our sample size was too small to adequately investigate the relationship between functional reorganization and clinical–demographic factors (i.e., duration of epilepsy, frequency of seizures, antiepileptic drug use, age and educational level), that have previously been shown to influence memory performance in mTLE (Lespinet et al., 2002; Motamedi and Meador, 2003; Oyegbile et al., 2004; Pai and Tsai, 2005; Thompson and Duncan, 2005). In the current study, we aimed to extend our understanding of network reorganization and mediation of WM performance in patients with mTLE: in particular, we wanted to assess whether between patient variations in effective connectivity were associated with relevant clinical and demographic factors (van Dellen et al., 2009; Vlooswijk et al., 2010a, 2011b; Voets et al., 2009). With this aim, we augmented our previous sample by studying more TLE patients with left HS (Campo et al., 2012) and with a special focus on between subject differences.

## Materials and methods

### Participants

Twenty patients (12 females; mean age 33.6 ± 7.32) with medically refractory MTL epilepsy undergoing presurgical evaluation at the Hospital Ruber Internacional were enrolled in this study. All patients underwent neurological examination, continuous video-EEG monitoring, and high resolution 1.5 T brain MRI. Patients were included in the study when clinical data, MRI and EEG findings suggested unilateral mesial temporal lobe epilepsy related to left HS. All patients had: i) seizures with typical temporal lobe semiology that were not controlled with antiepileptic drugs (AEDs) and ii) moderate to severe decreased volume (and abnormally increased T2 and FLAIR signal) of the left hippocampus on brain MRI. No seizure occurred within 24 h prior to the experiment. At the time of study patients were on AED treatment, including levetiracetam, lamotrigine, oxcarbazepine, carbamazepine, valproate, topiramate, zonisamide, clonazepam, lorazepam, either in monotherapy or multitherapy.

Twelve healthy volunteers (six female; mean age 31.58 ± 3.03) were recruited as normal controls for the study. Controls were interviewed and entered in the study if they met the following inclusion criteria: i) absence of a previous history of neuropathological or psychopathological conditions; and ii) no antecedent of drug or alcohol abuse. Participants were right handed according to the Edinburgh Handedness Inventory (Oldfield, 1971). All participants signed a consent form detailing the procedures of the study in accordance with the Declaration of Helsinki (1991) and guidelines from the local ethical committee. Demographic and clinical information is provided in Table 1.

### Stimuli and tasks

Participants performed a verbal-semantic WM task (Campo et al., 2005, 2009). The task involved the presentation of a stimulus array comprising four target words for 3000 ms. After a 2500 ms delay interval participants were presented with three consecutive probes comprising a semantic category name for 500 ms. A push-button response was required to indicate whether any of the target words belonged to the category probes. Thus, correct performance required subjects to maintain the target words in memory and generate a semantic categorization; ensuring a deep processing of probe words. There was an inter-stimulus interval of 500 to 700 ms. Match and no-match trials occurred with equal probability.

Concrete words were used, four to seven letters in length (5.62 ± 1.57) and of moderate frequency (Algarabel, 1996). A total of 120 trials were presented. The stimuli were projected through a LCD video-projector (SONY VPL-X600E), situated outside the shielded room, onto a series of in-room mirrors, the last of which was suspended approximately 50 centimetres above the subject's face and subtended a visual angle of 1–3° horizontally and 0.5° vertically.

### Data acquisition and analysis

MEG recordings were obtained using a whole-head neuromagnetometer comprising an array of 148 magnetometers (4-D 2500®, San Diego) housed in a magnetically shielded room. Electromagnetic signals were digitized continuously at 678 Hz and were band-pass filtered between 0.1 and 100 Hz. MEG data were submitted to an interactive noise reduction procedure to reduce environmental noise (4-D 2500®, San Diego). Data were analyzed using SPM8 (Wellcome Trust Centre for Neuroimaging, London; http://www.fil.ion.ucl.ac.uk/spm/). The continuous time series for each participant was processed with a Butterworth band-pass filter at 3–30 Hz and was then epoched off-line to obtain 900 ms data segments corresponding to − 100 ms to 800 ms peristimulus time. We analyzed epoched data during this period for each trial, for each participant. Trials including eye blinks or other myogenic or mechanical artifacts were removed using the thresholding criteria implemented in SPM8 (trials containing signal strength exceeding 3000 fT were excluded). Epochs were then baseline corrected from − 100 to 0 ms and then averaged.

### Dynamic causal modelling

**Fig. 1:**
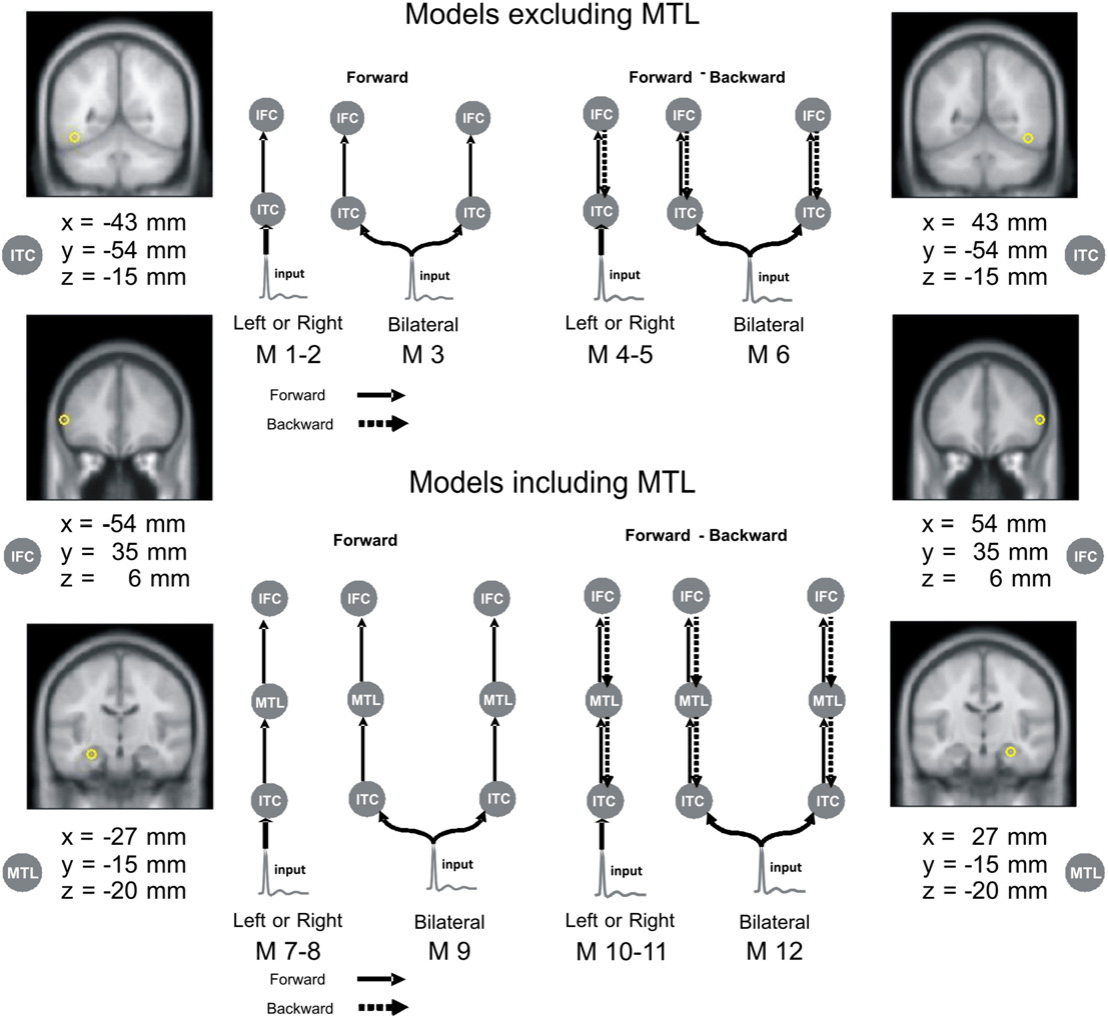
Model specification. This figure provides a schematic representation of the twelve models and their constitutive sources for the effective connectivity analysis. The brain regions comprising the network architecture, and their coordinates, are shown on coronal slices. IFC = inferior frontal cortex; ITC = inferior temporal cortex; MTL = medial temporal lobe. Stimulus extrinsic input is to ITC. Models differed in hierarchical levels (i.e. sources and extrinsic connections). Model sources could be unilateral (left or right), or bilateral. The upper row shows models without MTL and the lower row shows models with MTL. Arrows between the regions indicate the direction of the connections: ‘forward’ or ‘forward and backward’ (dashed lines).

DCM for evoked magnetic fields (ERF) allows for inferences about the neural networks or architectures that generate electromagnetic responses. These architectures are parameterized in terms of coupling within and between neuronal sources. Bayesian model comparison is used to compare different models or to find the model with the most evidence (Penny et al., 2004). This allows one to compare alternative hypotheses (models) of how measured data are caused (Friston, 2011). In our analyses, we first optimized the model and then compared the parameter estimates – under the best model – to test for group differences in effective connectivity. The sources or nodes of the network were specified using standard source localization procedures (i.e. multiple sparse priors; (Friston et al., 2008), and were therefore optimized for the particular subjects studied. Sources were then modelled as equivalent current dipoles (ECD), in a canonical brain (MNI) space, with prior mean location coordinates (*x*, *y*, *z*) at: ITC: − 43, − 54, − 15 (left), and 43, − 54, − 15 (right); MTL: − 27, − 15, − 20 (left) and 27, − 15, − 20 (right); and IFC: − 54, 35, 6 (left), and 54, 35, 6 (right). Our models differed in terms of number of sources, their laterality, and the type of directed connections (see Fig. 1 for details). All models were specified and inverted separately for each subject using a standard (Variational Laplace) scheme. Models were inverted using responses from stimulus onset to 800 ms poststimulus time. To select the most likely model we used Bayesian model selection (BMS) (Penny et al., 2004). The model with the highest evidence (i.e., the model with the best balance of accuracy and complexity) was considered the optimal model. We used a random-effect analysis (RFX) for comparing model-evidence; an approach that admits different models for different subjects and that is preferred when modelling “cognitive tasks that can be performed with different strategies” (Penny et al., 2010). All models were also compared at a single subject level (Penny et al., 2004). After selecting the optimal model, its subject-specific parameters were compared to test for between subject differences, using classical inference in the usual way (restricting the comparisons to parameter estimates that differed from their prior mean with a posterior confidence of 90%).

## Results

### Behavioral results

Performance was assessed in the verbal WM task in terms of correct hits for each stimulus set. We observed a mean accuracy level of 73.84% (SD = 10.27) in the control group, and mean accuracy of 59.07% (SD = 10.95) in the patient group. Group differences were analyzed by univariate analysis of variance (ANOVA) with Performance as dependent measure and Group (Patients and Controls) as a factor. Task performance was better for controls than for patients (F_1,30_ = 14.28, p < .001). The relationship between task performance and demographic (age, and length of education) and clinical variables (duration of epilepsy, frequency of seizures, and number of AEDs) was evaluated using multiple linear regression analysis. We found that level of education was significantly associated with task performance across subjects (*β* = .68, t = 5.40, p < .001; model R^2^ = .54, p < .001). Clinical variables did not significantly influence task performance (model R^2^ = .22, p > .20).

### Bayesian model selection

**Fig. 2:**
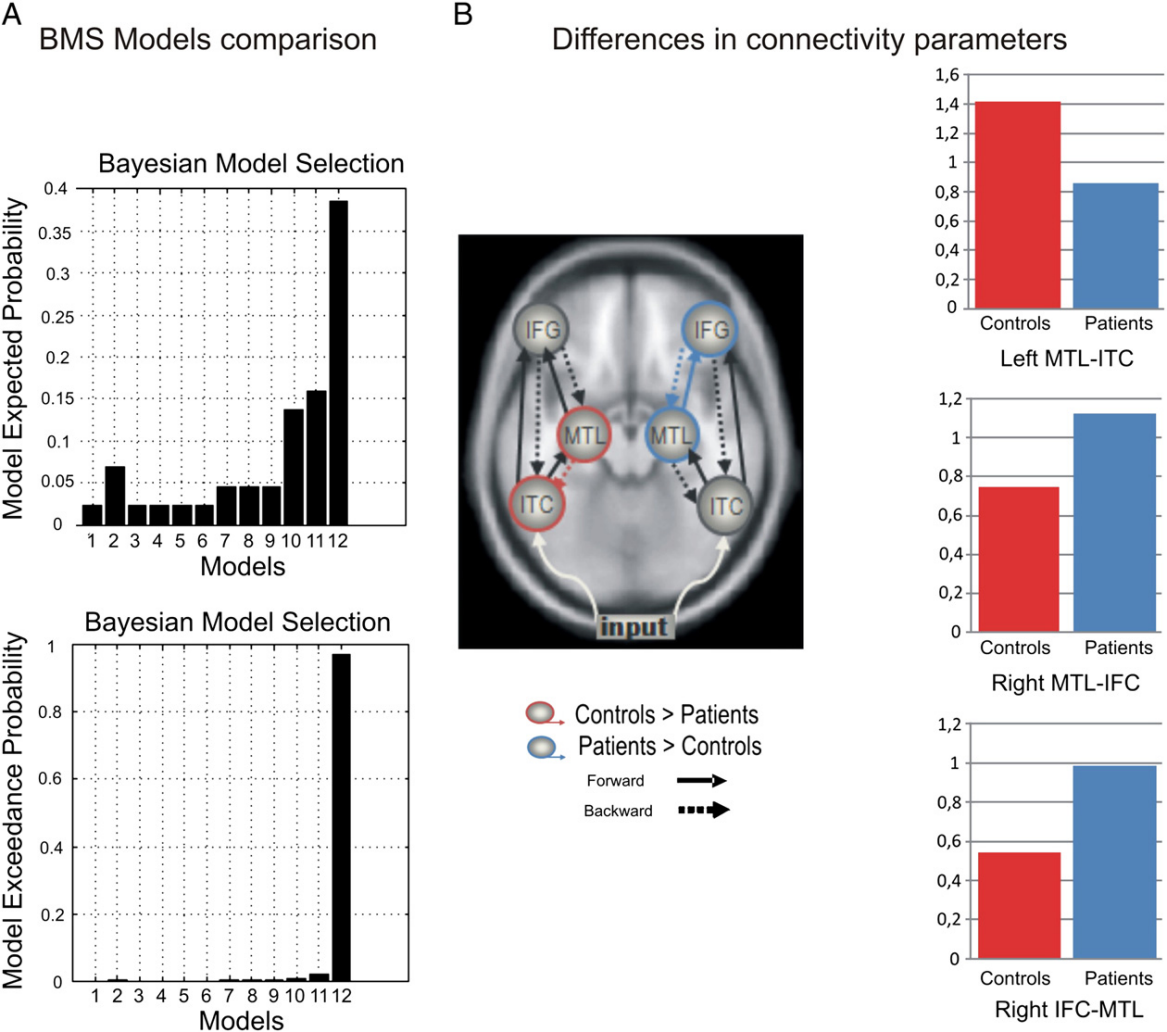
A) Bayesian model selection among the 12 models in Fig. 1. Random fixed effects (RFX) model comparison shown in terms of each model's evidence and exceedance probability. These results indicate that model 12 had the greatest evidence (exceedance probability = 0.975). B) The subject-specific parameters (with posterior probabilities of 90% or more of exceedingly prior mean) were selected to test for group differences. Red indicates ‘controls’ and blue indicates ‘patients’.

Model selection (RFX) revealed that the most likely model had bilateral forward and backward connections (with an exceedance probability of 0.975) (Fig. 2A). Model comparison was also performed for each participant individually. This confirmed that, for the majority of the patients (13 out of 20) and controls (7 out of 12), this model (M12) was superior to all other models.

### Between subject differences in connectivity

Group differences in effective connectivity strength were analyzed by a multivariate analysis of variance (MANOVA) with connectivity parameters as dependent measures and Group (Patients and Controls) as a factor. Effects were considered statistically significant when p < .05, after Bonferroni correction. Our analyses confirmed our previous report (in this extended sample) that the extrinsic backward connection from left MTL to left ITC was stronger in controls (Mean = 1.42; SD = 0.62) than in patients (Mean = 0.86; SD = 0.40) (F_1_,_30_ = 9.74, p < .005). In the right hemisphere, contralateral to the lesion, the backward connection from IFC to MTL was greater for patients (Mean = 0.99; SD = 0.44) than for controls (Mean = 0.54; SD = 0.20) (F_1_,_30_ = 10.57, p < .005). The reciprocal forward connection – from right MTL to right IFC – was also greater in patients (Mean = 1.12; SD = 0.46) as compared to controls (Mean = 0.75; SD = 0.36) (F_1_,_30_ = 5.92, p < .025). We performed a multiple linear regression analysis to characterize the independent influence of each of the demographic and clinical variables on connectivity parameters. Crucially, length of education showed a significant negative relationship with right backward IFC-MTL connectivity strength across subjects (*β* = − 0.60, t = − 4.72, p < .001; model R^2^ = .38, p < .005). We failed to detect a significant effect of clinical variables. To test whether the effect of education on connectivity was disease related or not, we used a median-split approach to identify two subgroups of patients with low and high levels of education. We then compared the strength of the contralesional IFC-MTL between the two educational levels and with that of the control group. We found that Low-educated patients showed stronger right IFC-MTL coupling than high-educated patients (t_18_ = 2.59, p < .01). Coupling strength was also higher in High-educated and Low-educated than in controls ([t_20_ = 1.81, p < .05] and [t_20_ = 4.91, p < .001], respectively). These results show that although connectivity strength in the contralesional hemisphere between IFC-MTL was influenced by the level of education, it was increased in the group of patients as compared to control subjects when level of education was controlled.

### Balance of connectivity between the ipsilesional and contralesional connections

**Fig. 3:**
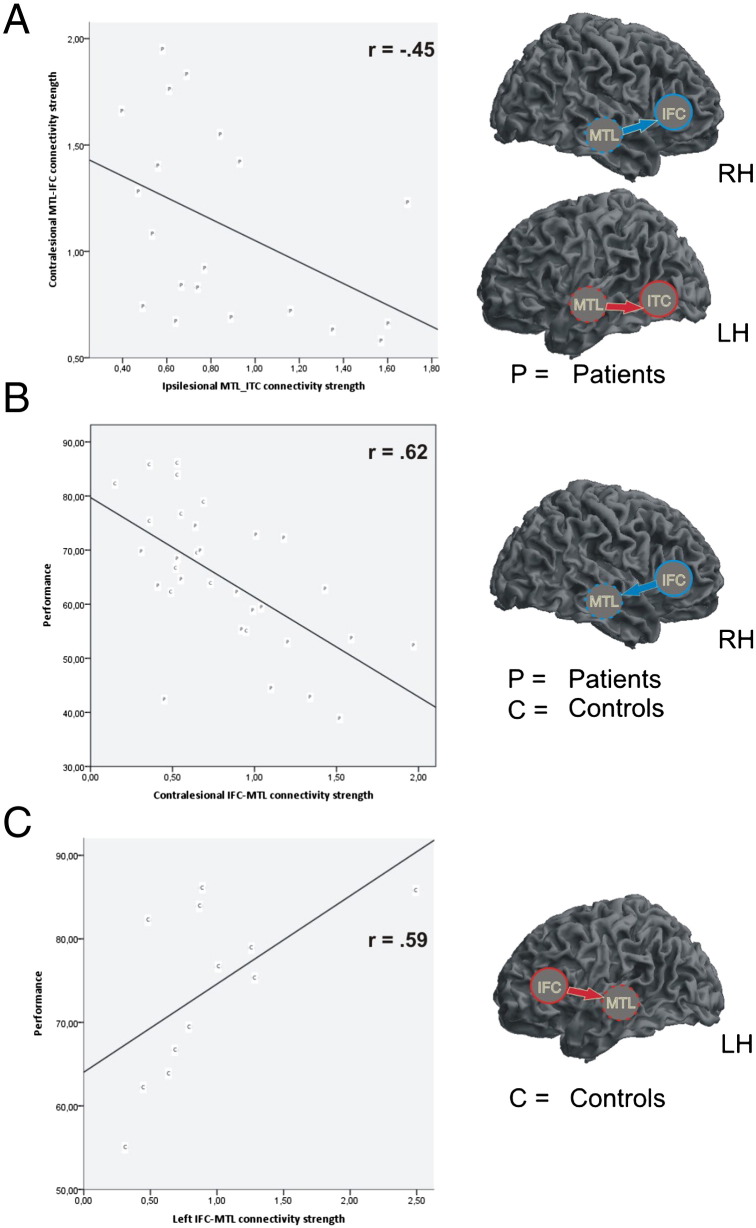
Scatterplots displaying the relationship between A) the connectivity from (ipsilesional) left MTL to left ITC and the strength from (contralesional) right MTL to right IFC in the group of patients; B) the backward connections from right IFC to right MTL were inversely related to task performance in both groups; and C) the backward connections from left IFC to left MTL and task performance in the control group.

In order to test our hypothesis (Campo et al., 2012) that contralateral enhancement of effective connectivity could reflect a ‘connectional diaschisis’, we computed the correlation of the connectivity strengths among regions in the WM network in patients where we found group differences. The coupling from left MTL to left ITC was negatively correlated with coupling strength from right MTL to right IFC (*r* = − .45, p = .049) (Fig. 3A). The correlation between left MTL-ITC and right IFC-MTL failed to reach significance (*r* = − .34, p > .10). A right preponderance in the backward MTL-ITC connection was also observed, although it did not reach statistical significance (t_19_ = 1.65, p = .058).

### Relationship between connectivity and task performance

We assessed the behavioral significance of group differences in effective connectivity by correlating the percentage of correct responses with the effective connectivity parameters. This analysis showed that the backward connections from right IFC to right MTL (in the non-lesioned hemisphere) were inversely related to task performance (*r* = − .62, p < .0005) overall subjects (Fig. 3B). We also observed a positive correlation between backward connections from left IFC to left MTL and task performance in the control group (*r* = .59, p < .05) (Fig. 3C). A left-right asymmetry in effective connectivity was found for this connection in controls, with a left predominance (t_11_ = 1.99, p < .05).

## Discussion

In this study we examined the impact of unilateral left hippocampal sclerosis on the reorganization of the functional architecture of the neural networks underlying verbal WM, and investigated whether this reorganization was influenced by relevant clinical and demographic factors. Using DCM for event related responses, we first established that the best model (over all subjects) involved a bilateral network comprising frontotemporal sources; such as ITC, IFC and MTL. These areas were interconnected through forward and backward connections (Fig. 1). By comparing specific network parameters between groups, we corroborated our previous findings (Campo et al., 2012) and found that patients displayed network adjustments that were characterized by a decreased backward connectivity from MTL to ITC in the ipsilesional hemisphere, and a bidirectional increase in IFC-MTL coupling in the contralesional hemisphere (Fig. 2). A number of novel findings were obtained regarding the interaction between network reconfiguration, WM performance, and clinical and demographic factors in mTLE. It was demonstrated that poorer WM performance and lower level of education were associated with a greater backward connectivity from IFC to MTL in the contralesional hemisphere (Fig. 3B). We also found that the decreased connectivity from MTL to ITC in the lesional hemisphere was related with a strongest forward connectivity from MTL to IFC in the non-affected hemisphere (Fig. 3A) (Bettus et al., 2009). These findings illustrate how unilateral HS induces not only local network abnormalities within the lesioned MTL, but more widespread effects in distal network interactions (Carter et al., 2012; Gratton et al., 2012), and that both are relevant to evaluate the network reorganization associated with HS.

Previous negative relation between memory performance and increased functional connectivity between hippocampus and diffuse areas of prefrontal cortex has been identified in amnestic mild cognitive impairment patients showing hippocampal atrophy (Bai et al., 2009). Likewise, a negative correlation between verbal memory performance and fMRI activation in the contralesional MTL have previously been observed in left mTLE-HS patients (Powell et al., 2007), and interpreted as an inefficient compensatory process. Therefore, the connectivity enhancement observed in the current study could be interpreted as a less efficient network adjustment (de Haan et al., 2012; Maccotta et al., 2007; Vlooswijk et al., 2011b). The decreased backward MTL-ITC connectivity in patients in the ipsilesional hemisphere induced by MTL damage is of relevance, as a top-down modulatory effect from MTL to content-specific regions in the ITC via rhinal cortex (Olsen et al., 2009; Schwindel and McNaughton, 2011; Vitay and Hamker, 2008) has been suggested as a mechanism for the reinstatement of specific information processed in those regions (Szatmary and Izhikevich, 2010; Woloszyn and Sheinberg, 2009). This interaction is proposed to be controlled by MTL based on short-term couplings from neural assemblies in MTL to corresponding assemblies in the neocortex (Cartling, 2001), and has been considered as a key neural process for WM encoding and maintenance (Fell and Axmacher, 2011). Coupling differences between groups could be signalling a process of structural degeneration associated with HS (Addis et al., 2007; Bettus et al., 2009; Frings et al., 2009; Liao et al., 2011; Vlooswijk et al., 2011b) leaving a less amount of recruitable neural assemblies available (Mormann et al., 2007), then leading to a reduced interaction between brain areas. Supporting this interpretation, greater preoperative functional connectivity between the hippocampus and the superior temporal gyrus was observed in patients who experienced a decrease in verbal learning postoperatively than for those who remained stable or improved postoperatively (Wagner et al., 2007). Furthermore, the negative relation of the strength of this connection with that in the contralesional hemisphere (i.e. MTL-IFC) could be viewed as an indicator of a pathological state (Maccotta et al., 2007). Consistent with this finding, a recent study demonstrated that, in a group of patients with mTLE, increases in basal functional connectivity in the non-epileptic MTL as compared to the epileptic one was the most specific marker of the epileptogenic zone (Bettus et al., 2010). Considered together, these results suggest that changes in these connections could quantify the functional integrity of the network (i.e. functional vs. dysfunctional) after localized damage to MTL (Doucet et al., 2012; Guedj et al., 2011; Maccotta et al., 2007).

Of the demographic and clinical seizure variables studied, only length of education was found to be associated with connectivity measures, and also with WM performance. Educational level has been previously reported to be related with structural connectivity measures in patients with TLE (Mueller et al., 2012), and has been considered a relevant variable affecting cognitive comorbidity in patients with TLE (Oyegbile et al., 2004; Pai and Tsai, 2005). The absence of effect of the other factors under consideration in this study (Bettus et al., 2009; Vlooswijk et al., 2010b) does not mean that no relation exists (Tracy et al., 2012; van Dellen et al., 2009; Vlooswijk et al., 2011b), and could be a function of the study sample. Additionally, as pointed by Vlooswijk et al. (2011a,b), factors cannot be considered as totally independent, as lower educational attainment could be related to a more disabling and severe epilepsy.

Finally, a greater left IFC-MTL coupling was associated with better performance in the control group. It is also important to highlight that an asymmetry was observed for this coupling in controls, with higher effective connectivity in the left hemisphere. These data support the relevance of frontotemporal interactions in human WM by showing that individual differences in task performance can be related to differences in the strength of frontotemporal connectivity (Gordon, 2011). Functional coupling between PFC and MTL is considered one of the core connections in the neural circuitry underlying WM (D'Esposito, 2007). Several studies have shown that increasing WM demands (Finn et al., 2010; Rissman et al., 2008) leads to a greater coupling strength between PFC and MTL structures. Additionally, recent studies have reported direct empirical evidence that associate structural and functional connectivity measures between the hippocampus and the PFC, which were correlated with better performance in a WM task (Cohen, 2011; Harms et al., 2013).

In summary, patients with mTLE-HS displayed a network reorganization selectively affecting connections involving the MTL that a wealth of evidence suggests are essential for WM (Poch and Campo, 2012). This reorganization was characterized by a decrease in effective connectivity involving MTL interactions within the lesional hemisphere, and an enhancement of effective connectivity parameters in the contralesional hemisphere between MTL and IFC, which were negatively related. Crucially, it was found that cognitive status was linked to specific network disturbances. Specifically, lower level of education and poorer WM performance were strongly associated with contralesional IFC-MTL connectivity strength. Thus, the induced network reconfiguration reflected altered functional interactions that represent compensatory, with varying degrees of efficacy (Maccotta et al., 2007; Masdeu, 2012), and pathophysiological mechanisms in response to MTL damage. These findings support the notion that network alterations underlie cognitive impairments in patients with mTLE-HS (Vaessen et al., 2012). In contrast, greater top-down IFC-MTL connectivity strength in the left hemisphere was related to performance in healthy controls. This study suggests that network dynamics and cognitive performance are related and that the evaluation of effective connectivity changes -that are remote from the site of lesion- could be used to assess the functional integrity of the WM network in mTLE-HS.

## Figures and Tables

**Table 1:** Demographic and clinical details of patients and controls.

	TLE (*n* = 20)	Controls (*n* = 12)
Age	33.60 (7.32)	31.58 (3.03)
Years of education	15.35 (1.98)	16.75 (1.14)
Duration of epilepsy (years)	20.40 (11.22)	
Age at epilepsy onset (years)	13.65 (8.67)	
Seizure freq. (per month)	3.50 (0.91)	
AEDs (number)	1.85 (0.62)	
1	30%	
2	55%	
≥ 3	15%	

AEDs = antiepileptic drugs.
